# From genes to behavior: placing cognitive models in the context of biological pathways

**DOI:** 10.3389/fnins.2014.00336

**Published:** 2014-11-04

**Authors:** Ignacio Saez, Eric Set, Ming Hsu

**Affiliations:** ^1^Helen Wills Neuroscience Program, Haas School of Business, University of California, BerkeleyBerkeley, CA, USA; ^2^Department of Economics, University of Illinois at Urbana-ChampaignUrbana, IL, USA

**Keywords:** gene pathways, computational models, neurogenetics, neuroeconomics, dopaminergic signaling, cognitive phenotyping

## Abstract

Connecting neural mechanisms of behavior to their underlying molecular and genetic substrates has important scientific and clinical implications. However, despite rapid growth in our knowledge of the functions and computational properties of neural circuitry underlying behavior in a number of important domains, there has been much less progress in extending this understanding to their molecular and genetic substrates, even in an age marked by exploding availability of genomic data. Here we describe recent advances in analytical strategies that aim to overcome two important challenges associated with studying the complex relationship between genes and behavior: (i) reducing distal behavioral phenotypes to a set of molecular, physiological, and neural processes that render them closer to the actions of genetic forces, and (ii) striking a balance between the competing demands of discovery and interpretability when dealing with genomic data containing up to millions of markers. Our proposed approach involves linking, on one hand, models of neural computations and circuits hypothesized to underlie behavior, and on the other hand, the set of the genes carrying out biochemical processes related to the functioning of these neural systems. In particular, we focus on the specific example of value-based decision-making, and discuss how such a combination allows researchers to leverage existing biological knowledge at both neural and genetic levels to advance our understanding of the neurogenetic mechanisms underlying behavior.

## Introduction

There is widespread interest in the application of formal computational models to connect behavior to its underlying biological substrates (Glimcher and Rustichini, 2004; Sugrue et al., 2005; Landis and Insel, 2008; Rangel et al., 2008; Behrens et al., 2009; Ebstein et al., 2010). At the neural level, we now have substantial knowledge of computational properties underlying a number of important domains of human cognition and behavior, and the set of brain regions that perform these functions (Glimcher and Rustichini, 2004; Landis and Insel, 2008; Rangel et al., 2008; Behrens et al., 2009; Ebstein et al., 2010). An intriguing question that has only recently become possible to address is the extent to which we can extend this understanding to uncover the genetic forces shaping and constraining these systems (Frank and Fossella, 2011; den Ouden et al., 2013).

This has important scientific and clinical implications. First, identifying mechanisms by which genomic differences lead to variations at cellular and neural circuit levels, resulting in changes in behavior and cognition, is an important step toward informing and improving the diagnosis and treatments of behavioral disorders (Glimcher and Rustichini, 2004; Sugrue et al., 2005; Landis and Insel, 2008; Ebstein et al., 2010; Insel, 2010; Kapur et al., 2012). In addition, the prospect that computational models can uncover not only computations at the circuit level, but also gene variation that influences these circuits, should substantially bolster the prospect that they have clinical utility (Meyer-Lindenberg and Weinberger, 2006; Rangel et al., 2008; Behrens et al., 2009; Montague et al., 2012).

However, despite the growing number of studies linking gene variation to complex behavioral traits in humans, comparatively few studies have attempted to link genotype data to behavioral phenotypes through the lens of computational models of behavior. This is even so in cases where existing models have shown considerable validity at both neurophysiological and molecular levels, as in the case of reinforcement learning models of reward-guided behavior (Schultz et al., 1997; Dayan and Niv, 2008; Doya, 2008; Frank and Fossella, 2011; den Ouden et al., 2013). One possible reason is these computational models, which are most often used in neuroimaging studies and therefore focus on capturing variation at the circuit level, are simply not well suited for capturing variation that operates on the developmental and evolutionary timescales (Bell and Robinson, 2011).

Here we argue that, on the contrary, computational models are useful precisely because they provide valuable mechanistic explanations at the intermediate neural levels so often absent in human studies linking genes, and behavior (Frank and Fossella, 2011). That is, because the effects of genetic and molecular mechanisms operating at longer timescales are necessarily mediated by neural mechanisms, computational models provide a framework through which we can unveil the impact of more distal effects of genes and molecules on the intermediate systems (Landis and Insel, 2008; Zhong et al., 2009; Bogdan et al., 2012).

Perhaps most importantly, when combined with emerging analytical approaches in genomics that enable researchers to focus on specific biological pathways and networks, these models allow behavior across different studies to be unified within a common biological framework. In doing so, this promises to move us beyond accumulating lists of significant gene-behavior pairings, and toward attempting to organize them in a unified and coherent mechanistic framework.

Here, we review analytical strategies and concepts to enable a biologically informed characterization of neurogenetic mechanisms underlying value-based decision-making in humans, and describe how to integrate them with computational principles that are beginning to emerge from the burgeoning neuroimaging literature tying formal mathematical models to choice behavior at the level of neural circuits. Our goal is to propose a new analytical strategy that combines computational models and gene pathways that can be used to unveil mechanistic relationship between genetic variants and behavior. To this end, we will review the foundations of the approach: (1) computational models of behavior, and how they can be used as cognitive phenotypes, and (2) the use of gene pathways as a strategy to balance the competing demands of interpretability and discovery in the analysis of human genetic data; finally, we will review a prior application of these principles (Set et al., 2014) as a case study that illustrates the fruitful combination of these two approaches.

## Genetics of human behavior

Two main research strategies exist for the identification of genes associated with heritable traits—candidate gene approaches and genome-wide association (GWAS) approaches (Yang et al., 2010; Flint and Munafo, 2013) (see Box 1 for glossary of genetic terms). While linkage studies are also available, we focus on association studies in this perspective as they are increasingly the primary tool in the case of human studies (Sabb et al., 2009). First, in candidate gene studies, one or a small number of gene variants with known effects on the protein structure or expression are used to detect genotype-phenotype associations (Flint et al., 2001; Flint and Munafo, 2013). These studies are typically motivated by prior knowledge of biological mechanisms underlying the physiology of a certain trait. In GWAS studies, this goal is achieved using all gene variants across the entire genome, which are independently tested in a hypothesis-free manner (International Schizophrenia Consortium, 2009; Rucker et al., 2011).

Box 1 **Some prerequisites for understanding neural and genetic studies of behavior.**

- **Allele**: One of two or more forms of a gene, located on a specific position on a chromosome.
- **Candidate gene studies**: Studies that focus on association of pre-specified genes of interest, typically based on prior knowledge, and phenotypes.
- **Genome-wide association studies (GWAS)**: Studies that aims to find associations by scanning common genetic variation in the entire genome in hypothesis-free manner.
- **Gene pathway**: A group of functionally related genes that mediate a particular biological process, e.g., DA functioning.
- **Linkage Disequilibrium**: Extent to which alleles are correlated due to common inheritance. Alleles of nearby genes are typically in high linkage disequilibrium.
- **Minor allele frequency (MAF)**: The frequency at which the *least common* allele occurs in a given population. Typically alleles with MAF below 5% or 10% are excluded from the study.
- **Single Nucleotide Polymorphism (SNP)**: In genetics, a difference in DNA sequence among individuals. A common form of a genetic polymorphism is a SNP, which occurs when a nucleotide—A, T, C or, G—differs between individuals. The human genome contains millions of SNPs. Below are a list of common types of polymorphisms.
  ◦ **Exonic mutation**: Polymorphisms in gene region that remains present within the final mature RNA product.
  ◦ **Synonymous mutation**: Exonic mutations that do not modify the protein encoded by the gene. Previously thought to be silent but now known to have potential effects on transcription, splicing, mRNA transport, and translation (Sauna and Kimchi-Sarfaty, 2011).
  ◦ **Non-synonymous mutation**: Exonic mutations where the protein encoded by the gene is modified.
  ◦ **Intronic mutation**: Region within a gene that is removed by RNA splicing while the final mature RNA product of a gene is being generated. Previously thought to be silent but now known to have potential effects on splicing accuracy and translational efficiency (Cartegni et al., 2002).
  ◦ **Untranslated region (UTR)**: Region directly adjacent of coding region of the gene, important for regulation of RNA translation.
  ◦ **Intergenic regions**: Stretches of DNA sequences located between genes. Most variants in this region have no currently known function, but some are thought to have regulatory functions. In humans, intergenic regions comprise about 80%–90% of the genome.

**Figure 1:**
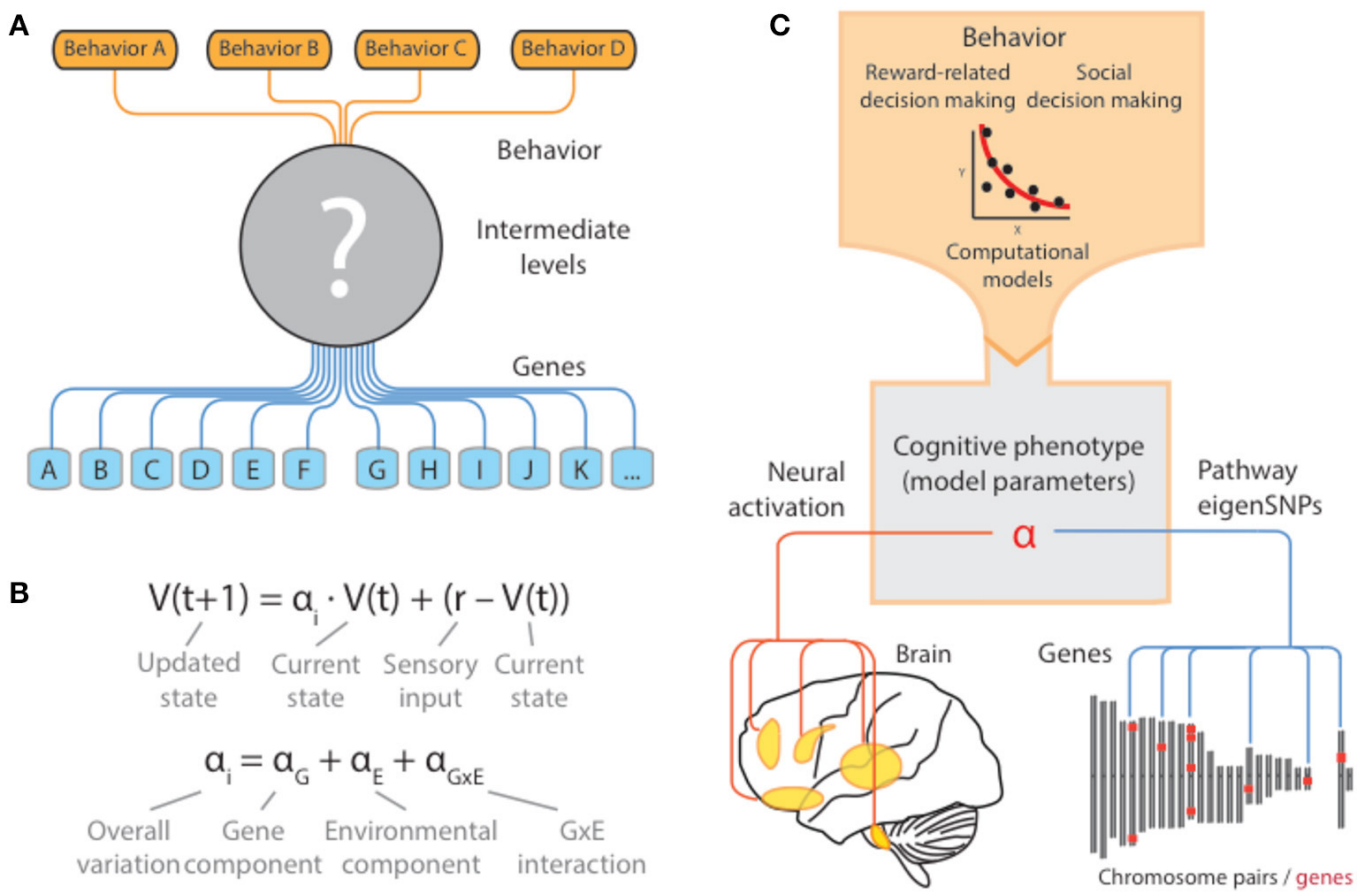
**Cognitive models as quantitative descriptions of putative intermediate mechanisms**. **(A)** For most human behaviors of interest, the intermediate neural, synaptic, and molecular mechanisms are far from clear. As a result, studies of the genetic basis of these behaviors are forced to directly examine the effects of the chosen genotype onto behavior, without consideration of the ways in which genetic variation propagates through and constrains these intermediate levels. **(B)** Computational models provide a principled way in which complex patterns of behavior can be quantified and reduced to a lower-dimensional space via the set of parameters governing the computations. Variation of the parameters in the population can be related to underlying genetic variation and other inter-individual factors (i.e., environmental) and interactions. In the example, a schematic of a simple reinforcement learning model is presented where the parameter α _i_ governs the extent to which an individual organism is sensitive to more recent rewards relative to past ones. This parameter in turn can be thought of as an intermediate cognitive phenotype that is under the influence of genes, environment, and their interaction. **(C)** When validated at the neural level, these models can serve as quantitative descriptions of the missing intermediate mechanisms through which genes exert their influence on behavior. In this sense, model parameters are equivalent to cognitive phenotypes and can act as a nexus that mechanistically connects different biological levels underlying behavior.

Despite the rapid growth of studies based on these approaches, and the accumulation of gene markers implicated in behavior, findings from these studies have been subject to widespread skepticism about their (i) reliability, and (ii) ability to inform us about the genetic architecture underlying behaviors and disorders where they are affected (Figure 1A) (Hart et al., 2013). At least in the case of human behavior, many behaviors of interest relate to highly human-specific activities that are the result of complex social, cognitive, and cultural influences. Thus, even in cases where candidate genes are carefully motivated and have clear biological implications, their connection to basic cognitive processes underlying the trait of interest can be unclear (Figure 1A) (Flint et al., 2001; Reuter et al., 2011; Flint and Munafo, 2013).

To use a concrete example, consider a previous study finding that voting propensity is associated with serotonin gene polymorphisms, specifically alleles in the MAOA and SERT (Fowler and Dawes, 2008) (Box 1). Although such studies provide valuable insights into possible biological substrates of an important feature of modern human civilization, a vast gap exists between the functions of these genes on the one hand, and the act of voting in an election in a modern Western democracy.

As the authors of the study point out, even taking genetic associations identified in the study as given, the nature of the genetic contribution remains far from clear (Fowler and Dawes, 2008). First, the identified polymorphisms may play a role in promoting prosociality, but it could also be related to aggression. It may increase the sense of satisfaction one derives from fulfilling a civic duty. It may increase the strength of desire for expression. It may be part of a broad constellation of personality traits. This is only a partial list of the possible ways that serotonin genes might influence voting propensity.

Perhaps more importantly, the lack of mechanistic insights has contributed to a fragmentation that impedes the accumulation of knowledge critical for scientific advancement. A central question, therefore, is whether it is possible for genetic studies of behavior, like those in morphology or simpler types of phenotypes, to trace through the complex biological pathways connecting genes and behavior in a way that makes it possible to integrate diverse behavior-genotype associations in a biologically based framework.

## Cognitive models as candidate mechanisms

Note that in all the above cases, the key question is how to relate and map diverse behavioral phenotypes to a more constrained set of intermediate cognitive phenotypes (Houle et al., 2010; Rasetti and Weinberger, 2011; Bogdan et al., 2012). That is, a crucial step in overcoming these hurdles is to reduce the distal behavioral phenotype to a set of molecular, physiological, and neural processes that render them closer to the actions of genetic forces. In the case of model organisms we have the ability to interrogate these molecular and neural mechanisms directly, but most are unavailable in humans due to their invasive nature.

At least in the case of the brain, our understanding has been transformed by recent applications of formal computational models that connect behavior to their underlying neural circuitry (Schultz et al., 1997; Montague et al., 2004; Behrens et al., 2009; Maia and Frank, 2011). In a number of cases, these models have been shown to have considerable validity at both behavioral and neural levels (O'Doherty et al., 2007; Rangel et al., 2008). For example, the basic temporal difference model is able to explain a variety of reward-guided behavior using a single parameter governing the strength of impact of the reward prediction error on future behavior (Figure 1B) (Schultz et al., 1997; Montague et al., 2004). At the neural level, although details regarding interpretation remain debated (Berridge, 2007), substantial evidence points to a key role of midbrain dopaminergic neurons in carrying a quantitative signal guiding choice behavior, which can be captured using both neurophysiological evidence in model organisms and neuroimaging evidence in humans (Dayan and Niv, 2008).

At the genetic level, then, cognitive models provide a principled way in which complex patterns of behavior can be quantified and reduced to a lower-dimensional space via the set of parameters governing the computations. Variation of the parameters in the population can be related to underlying genetic variation, and other inter-individual factors (i.e., environmental), and interactions (Figure 1C). This parameter in turn can be thought of as an intermediate cognitive phenotype that is under the influence of genes, environment, and their interaction.

In an early example of this approach, (Frank et al., 2007) investigated how genetic polymorphisms in candidate genes affected reward and avoidance learning in humans. Using a cognitive model that captures distinct computational components connected to reward and avoidance learning, the authors found that variation in different dopaminergic genes, specifically DARPP-32, DRD2, and COMT, were associated with separate parameters governing reward and avoidance learning. Importantly, these findings can be directly connected to our knowledge of how these genes relate to dopaminergic functioning. For example, both DARPP-32 and DRD2 are thought to affect primarily striatal, as opposed to prefrontal, dopamine (Missale et al., 1998), whereas the reverse is true for COMT (Männistö and Kaakkola, 1999). The fact that striatal dopamine genes affected the speed of learning is notable as it is consistent with a broad class of neurophysiological and neuroimaging work in both human and animal studies.

For example, associations of D2 receptor gene variation to behavior can be linked to its potential effects on striatal D2 receptor density, which are then linked to systems-level changes that translate to changes in behavior. Importantly, the predictions of this working model can be tested using pharmacological manipulation, PET imaging, or via invasive methods using model organisms. In contrast, such a systems approach would be considerably more challenging in distal phenotypes such as voting behavior.

Taken together, connecting genes to computational models therefore would help to address a key limitation in many studies of genetic basis of behavior (Figure 1C) (Frank and Fossella, 2011; den Ouden et al., 2013; Set et al., 2014). Importantly, a focus on mechanisms can advance existing conversation from one focused on “gene-hunting,” with a goal of accumulating highly significant polymorphisms regardless of their functional importance (or “behavior hunting” in the case of candidate genes, where one seeks to accumulate a list of behaviors regardless of their interdependence), to one focused on mechanism and the phenotype of interest.

## Gene pathways

**Figure 2:**
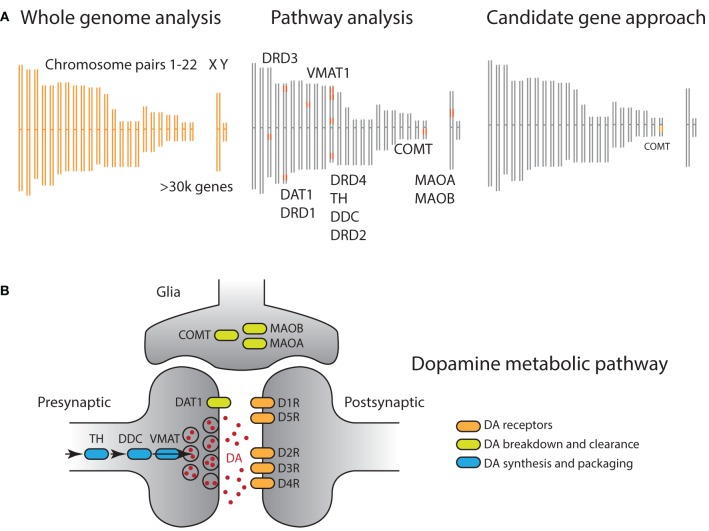
**(A)** Comparison of genomic analysis approaches illustrated on a DNA schematic, including 22 pairs of autosomal and sex chromosomes. Orange shaded regions indicate genetic materials used for analysis. (Left) GWAS analyses assess association of phenotype of interest with all sequenced SNPs, often hundreds of thousands, independently. Hence all chromosomes are shaded orange. (Right) On the other side of the technical spectrum, candidate gene approaches focus on a single polymorphism, often well motivated by prior biological data. In this example, the non-synonymous rs4680 SNP of the COMT gene is selected. (Middle) A pathway approach offers a compromise, where prior biological information is leveraged to define a set of genes, organized around a biological process. In this example, all genes whose products have an impact on dopaminergic neurotransmission are selected. **(B)** Dopamine metabolic pathway captures the biological process involved in neurotransmission, including dopamine synthesis (blue), dopamine signal transduction (orange), and dopamine transport and clearance (green). In principle, genes that regulate/act on these dopaminergic genes can also be included, although we do not include them here as they have broad functions in the nervous system.

Despite these promising features, candidate mechanisms are not by themselves sufficient to overcome the formidable challenges arising from the inherent complexity of genomic data. First, the sheer size of modern gene array data have resulted in a situation where it is often the rule rather than the exception that significant gene markers have little direct relationship to plausible biological mechanisms (Figure 2A). For example, a recent study (Rietveld et al., 2013) identified a genome-wide significant SNP that is significantly associated with a complex and distal phenotype, academic achievement; however, this SNP is not located in the proximity of any genes which might mediate its biological effect, and so how the effect comes to be is unclear even if we had a precise cognitive model of academic achievement. That is, even when there are candidate mechanisms available, the associated gene markers often have no discernible relationship with the mechanism.

Second, genes do not function independently but within biological pathways, and they interact within biological networks (Figure 2B) (Wang et al., 2007; Ramanan et al., 2012). In particular, the accumulation of weak but coordinated effects arising from multiple alleles within specific biological systems is increasingly thought to be an important source of phenotypic variation. The fact that both GWAS and candidate gene studies focus on individual genotype markers poses a challenge for them to detect subtle effects distributed across the genome (Wang and Abbott, 2008). This point is particularly crucial as it is now widely accepted that common alleles, including those used in candidate gene studies, exhibit modest effect sizes. As such, the statistical approach of treating individual alleles as independent results in a potentially serious loss of power by ignoring the underlying biological structure.

In recent years, studies that strike a middle ground, using so-called pathway approaches, are becoming increasingly popular (Figure 2A and Box 1) (Wang et al., 2007, 2010; Yaspan and Veatch, 2011; Ramanan et al., 2012). A genetic pathway consists of a group of functionally related genes that mediate a particular biological process, e.g., DA functioning (Figure 2B). Each gene along the pathway encodes a protein that carries out a specific biological function. For example, the DAT1 gene encodes the dopamine transporter (DAT), whose function is to remove dopamine from the synaptic cleft, thus terminating the signal of the neurotransmitter. Although these pathways are abstractions of complex biological process that have no discrete start or end points, they have been invaluable to researchers as they capture and organize our knowledge in a parsimonious and tractable manner.

The pathway approach addresses these issues by limiting our search to a set of genes underlying a specific biological process, thereby improving the interpretability of potential results (Wang et al., 2007, 2010; Yaspan and Veatch, 2011; Ramanan et al., 2012). For behavior, there are a number of molecularly defined pathways that are suitable as candidates based on previous anatomical, pharmacological and physiological studies in both humans and animals: neuromodulatory pathways (serotonergic, dopaminergic, noradrenergic, etc.), hormonal and, neuropeptide pathways (oxytocin, vasopressin), synaptic plasticity related pathways, growth factors such as neurotrophins (BDNF, NT-3, NT-4, etc.), and transcription factors, to name a few.

In particular, because a pathway approach fosters a view centered on biological processes, as opposed to individual polymorphisms, statistical inference can be made at multiple level of analysis, from SNP, to gene, to pathway, in a way that can adapt to the particular question, but without being completely unconstrained as in GWA studies (Chen et al., 2010; Ramanan et al., 2012). For example, compared to previous studies making inferences at the level of individual SNP or VNTR, in Set et al. [36] we considered the combined impact of all common polymorphisms within individual DA genes. With larger sample sizes, it is possible to compare whole pathways with hundreds of variants, as have been done in a number of disease studies.

## Case study: connecting cognitive models to gene pathways

Given the number of analytical steps involved in our proposed approach, we give in this section a detailed step-by-step guide to conducting pathway studies of cognition and behavior. To fix ideas we will use the specific example of a recent study by Set et al. (2014) that applied dopaminergic pathways to strategic learning.

### Phenotype

**Figure 3:**
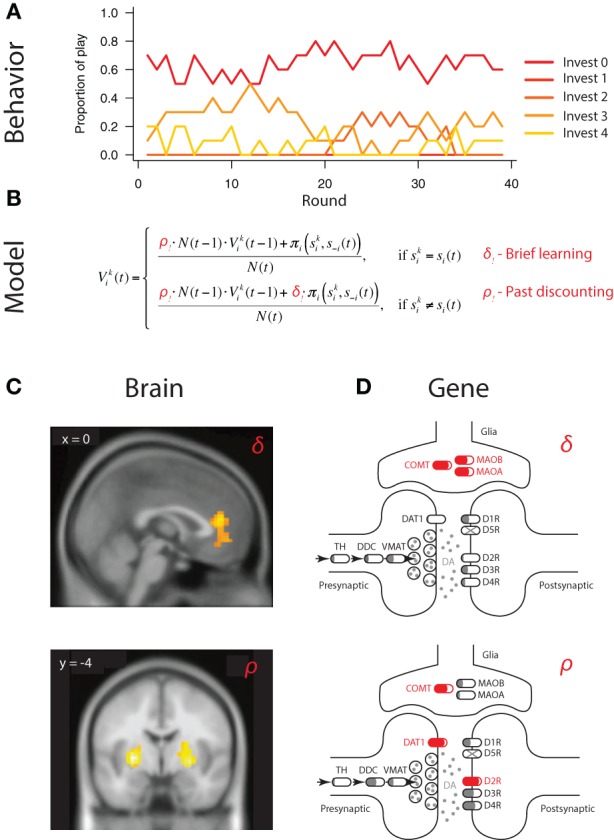
**Mapping neural and genetic correlates of strategic learning**. **(A)** Choice behavior in economic games provides basic material to characterize neural and genetic correlates of behavior. In this example, subjects make sequential choices over 240 rounds of a multi-strategy competitive learning paradigm, the patent race. **(B)** Trial-by-trial variation in behavior is captured by a model—experience weighted attraction (EWA)—containing two parameters governing two distinct aspects of strategic learning. (i) Belief learning parameter δ that captures the degree to which participants anticipate and respond to the actions of others, and (ii) learning rate parameter ρ captures the strength of past experiences on behavior. Individual differences, i.e., person-by-person variation, is captured by different parameter values of δ_*i*_ and ρ_*i*_ for participant i. **(C)** Neural circuits subserving specific computations can be mapped using outputs of the calibrated model outputs at a trial-by-trial level. In the example, belief learning signals were localized to mPFC activity, whereas reinforcement learning signals to striatal activity. Adapted from Zhu et al. (2012). **(D)** Genetic influence on behavior can similarly be mapped by connecting gene variation in the dopaminergic pathway to intermediate phenotype, captured by parameter variation at the individual level. In the example, variation in belief learning δ_*i*_ is significantly associated with variation in genes responsible for dopaminergic degradation (COMT, MAOB, MAOA), which govern dopaminergic levels in the prefrontal cortex but not striatum. In contrast, variation in learning rate ρ_*i*_ is significantly associated with variation in genes highly expressed in the striatum (DAT1, DRD2), but not prefrontal cortex. Interestingly, COMT variation is also associated with learning rate. Adapted from Zhu et al. (2012) and Adapted from Set et al. (2014) should be made consistent.

Strategic learning refers to decisions made in the presence of competitive or cooperative intelligent agents, where, in addition to learning about rewards and punishments available in the environment, agents need to also anticipate and respond to actions of others competing for the same rewards (Figure 3A) (Fudenberg, 1998; Hofbauer and Sigmund, 1998). Specifically, Set et al. (2014), applied the well-established experience weighted attraction (EWA) model to reduce individual variation in competitive winner-take-all paradigm to two key parameters capturing (1) the degree to which players are sensitive to actions of others, captured by δ, and (2) learning rate or sensitivity of players to more recent observations relative to past ones, captured by ρ (Figure 3B) (Sutton and Barto, 1998; Camerer, 2003; Zhu et al., 2012).

Importantly, this computational characterization of behavior was able to capture trial-by-trial variation in fMRI BOLD activity of players during game play (Hsu and Zhu, 2012; Zhu et al., 2012). Specifically, whereas the medial prefrontal cortex was found to respond selectively to belief-based inputs and reflected individual differences in degree of engagement of belief learning, striatal activity was correlated with both reinforcement and belief-based signals, suggesting possible convergence of these signals in the striatum (Figure 3C) (Zhu et al., 2012).

### Pathway selection

First, given the phenotype of interest and candidate cognitive model, one needs to determine the appropriate pathway involved. One option is to select a set of genes that are related to a specific biological function, such as neurotransmission. For many behavioral or cognitive processes, neuromodulatory systems such as dopamine and serotonin are particularly attractive targets (e.g., Figure 2B).

In the case of strategic learning, dopaminergic mechanisms are a natural candidate owing to the involvement of reward learning processes. Moreover, DA transmission is known to exhibit remarkable regional variation in expression levels of genes coding for the set of enzymes, receptors, and transporters involved in DA functioning (Pierce and Kumaresan, 2006; O'Connell and Hofmann, 2012) (Figure 2B). In the prefrontal cortex, where DAT1 expression is low, genes regulating enzymatic breakdown, in particular COMT and to a lesser extent isoforms of the MAO genes, are important determinants of DA flux (Nemoda et al., 2011). In contrast, these genes have much less impact on striatal DA levels, where DAT1 expression is high (Frank and Fossella, 2011). On the receptor side, regional variation results from distribution of DA receptor types (Missale et al., 1998). Receptors of the D1 family, D1 and, D5, are expressed throughout the brain. In contrast, receptors in the D2 family exhibit more regional specificity: D2 receptors are expressed primarily in the dorsal striatum, D3 receptors in the ventral striatum, including nucleus accumbens but less so in dorsal striatum, and D4 receptors in the frontal cortex and limbic regions (Missale et al., 1998).

Another popular technique is to use gene ontology annotations, such as the Gene Ontology (GO) database (Harris et al., 2004). A third option is to select genes that are expressed at a given developmental time in brain areas that are known or suspected to be implicated in said processes. Yet many others are possible, and we are only beginning to appreciate how to best divide the complex set of molecular and cellular processes in ways that shed light on cognitive processes.

Because the underlying biological processes have no real starting or ending points, the pathway definitions require decisions that trade off between coverage and interpretability. For example, for neurotransmitter-centered pathways, the focus point is the locus of action of the neurotransmitter, i.e., the neurotransmitter-receptor interaction in the synaptic cleft. From that pivot point, sets of genes that are involved in neurotransmitter synthesis, signal transduction, and signal degradation form the core of the pathway, which can then be concentrically expanded to include secondary messengers in the postsynaptic side, regulatory elements such as kinases and phosphatases, transcription factors, etc. The cost of such an expansion is a loss of statistical power and biological interpretability; for example, secondary messengers are promiscuous and are typically activated in response to activation of numerous membrane receptors, a characteristic akin to the pleiotropy of genetic effects.

### Assigning data elements to genes

Once the gene set underlying the pathway is determined, the set of data elements, whether SNPs, variable number of tandem repeats, or copy number variations (Box 1), must be decided. Due to the current technical capability and low cost of SNP sequencing, the former is by far the most common. All SNPs located in known coding or regulatory regions are typically analyzed, since they offer a straightforward connection to the biological effects of the genetic variation, mediated by changes in protein sequence. However, to capture possible regulatory variations, all SNPs within the coding region of the gene (both exonic and intronic) as defined by current genomic atlases may be included, as was the case in Set et al. (2014). Furthermore, upstream or downstream SNPs can have regulatory functions such as effect in transcriptional or translational efficiency, and may also be included.

### Dealing with linkage disequilibrium

Genes often contain multiple SNPs. Due to their physical proximity, they are often co-inherited and thus variation in them is typically correlated, an effect called linkage disequilibrium (LD) (Box 1). Analyzing each of these as an independent factor inflates the multiple comparison problem, and therefore statistical methods have been proposed to deal with this issue, such as principal component regression (PCR) (Wang and Abbott, 2008).

Specifically, this approach uses the first few principal components (PCs), so-called eigenSNPs, computed from the sample covariance matrix of SNP genotype scores as regressors, and has been used in a number of previous gene expression and SNP marker studies (10). For example, in Set et al. (2014), 4 eigenSNPs contained 91% of the variation in the COMT gene, from an initial set of 17 SNPs that exceeded an MAF threshold of 0.1.

Compared to traditional candidate gene approaches, this multilocus approach can be used to detect association between a phenotype and groups of SNPs (genes), and is more efficient when there exists weaker but coordinated effects arising from multiple SNP markers. Other solutions, such as shrinkage methods including LASSO and random forests, have been developed but increase the computational burden substantially (Bridges et al., 2011).

### Combining pathways and models

Once inter-subject genetic (through pathway analysis) and phenotypic (through computational models) variability have been assessed, they must be mapped onto one another. A multiple linear regression of genetic variation on estimated parameter values offers a simple way of doing this. Effectively, optimal weights for each piece of genetic variation (SNP, eigenSNP, etc.) are assigned to explain as much of the variation in parameter space as possible (Wang and Abbott, 2008).

For example, in Set et al. (2014), this involved allowing each parameter (e.g., δ) of the model to vary according to the set of associated eigenSNPs of each gene in the DA pathway. In the case of the COMT gene, this included the addition of four additional parameters {δ_1_, δ_2_, δ_3_, δ_4_} corresponding to the four eigenSNPs of the DAT1 gene, in addition to the population (mean) parameter δ. Intuitively, this analysis asks the question of whether inclusion of genetic information can improve statistical fit of the model by capturing individual differences.

At this stage, nuisance regressors that are known or suspected to impact the behavior under study can be included. For example, inclusion of the first 10–20 whole-genome principal components is an effective way of controlling for population stratification (Price et al., 2006).

### Assessment of significance

Although asymptotic tests are possible in this approach, potential violations of standard assumptions have led to the widespread use of permutation tests, which requires a weaker set of assumptions to be valid (Wang et al., 2010; Winkler et al., 2014). Here, the null distribution is created by shuffle the gene-behavior pairings, such that the observed association has to be significantly higher than that of a “random” genome (Wang et al., 2010; Winkler et al., 2014).

Alternatively, if one has access to GWAS data, one can compare the association in a particular gene to comparison “null” genes outside of the pathway that possess similar statistical properties (e.g., same number of SNPs that reduce to similar number of eigenSNPs). In Set et al. (2014), this is referred to as the “empirical *p*-value,” to distinguish from the permutation *p*-value. Importantly, because these genes are selected because of a hypothesized negative relationship (e.g., genes that do not express in the CNS), they provide a highly useful negative control with which to dissociate candidate pathways against null pathways.

### Biological interpretation of results

In the past, a significant hurdle existed in attempting to connect gene association findings to intermediate neural mechanisms. In the case of Set et al. (2014), restricting attention to the gene level and pathway alleviated potential interpretational issues considerably. First, the fact that belief learning processes engaged primarily medial prefrontal cortex accord well with the associations between belief learning parameter δ and variations in the COMT, MAOB, and, MAOA genes (Figure 3D). All three are genes implicated in dopamine catabolism and are responsible for regulating dopaminergic levels in the prefrontal cortex. In contrast, learning rate ρ was found to be significantly associated with variation in striatal genes DAT1 and DRD2 (Figure 3D). Overall, these findings raise a number of interesting questions regarding the anatomical specificity of the genetic effects, which can be tested in imaging genetic studies. For example, an interesting question is whether the COMT effect on learning rate is exerted through prefrontal DA or its indirect effects on striatal dopamine, as has been reported in previous imaging genetics findings (Dreher et al., 2009).

### Validation and followup

One important drawback of including all polymorphisms is that the functionality of the identified polymorphisms can be obscure. For example, of the 143 common SNPs in Set et al. (2014), only one, the extremely well-studied rs4680, is associated with a change in protein structure. The rest were either synonymous mutations or resided in intronic or untranslated regions. In recent years, however, there are a growing number of computational methods available to gain further insight into these potential biological functions. They rely on identifying sequences with known biological effects in the DNA sequence including and surrounding SNPs of interest. The SNPInfo web server (http://snpinfo.niehs.nih.gov), for instance, provides a web interface where multiple SNPs can be queried to obtain information about their potential biological effects for SNPs located in coding (protein sequence changes, changes in stop codons) and non-coding (transcription factor binding sites, splicing regulation, miRNA binding sites, etc.) regions.

In addition to mining existing data, new data can be acquired to gain insight into the nature of the association. For example, in the case of polymorphisms that putatively result in changes in protein concentration, what is the association between protein levels and the behavioral effect? Imaging genetics approaches can be used to gain further insight into the mechanisms whereby a genetic change affects neural mechanisms underlying a cognitive phenotype (Hariri et al., 2006; Klein et al., 2007). Pharmacological manipulations can further be carried out to demonstrate the causal involvement of the identified molecular mechanism. Although not all genes can be targeted, in the case of neural pathways there are a variety of drugs that have been applied to the study of behavior which affect different neurotransmitter systems such as dopamine, serotonin, neuropeptides (e.g., oxytocin) (Kosfeld et al., 2005; Pessiglione et al., 2006; Crockett et al., 2008). For the cases in which a more detailed examination is warranted or for which no pharmacological manipulation is possible, animal models can be used to investigate the impact of a single gene (e.g., gene knockouts, gene knockdowns).

## Conclusion

In contrast to phenotypes such as morphology, behavior has always presented special challenges for biological studies because of its temporal nature and context dependence (Houle et al., 2010). In the case of human behavior, the situation is even more challenging as many behaviors of interest relate to highly human-specific activities that are the result of complex social, cognitive, and, cultural influences (Bilder et al., 2009; Houle et al., 2010).

At the neural level, recent applications of functional neuroimaging, combined with formal economic models, have greatly expanded our understanding of the neurocognitive processes underlying complex behaviors, such as decision-making in strategic environments (Behrens et al., 2009; Burke et al., 2010; Hsu and Zhu, 2012; Zhu et al., 2012). At the same time, recent technical advancements have significantly advanced our knowledge of human genetic variation and the location and impact of human genetic polymorphisms.

Despite such progress, however, there has been surprisingly little attempt to connect and cross-pollinate these different levels in ways that emphasize the relative strengths of each approach while minimizing their weaknesses. In this perspective, we described an approach focusing on specific biological processes in ways that relate systems of functionally-related genes to putative mechanistic models of behavior (Wang et al., 2010; Yaspan and Veatch, 2011; Ramanan et al., 2012). Specifically, this involves linking, on one hand, working models of neural computations carried out by local circuits (Frank and Fossella, 2011), and on the other hand, the set of the biochemical processes that are carried out by genes (Wang et al., 2007; Ramanan et al., 2012).

Clinically, a better integration of genetic and neural data is an important step toward improving diagnosis and treatment of neuropsychiatric disorders (Gottesman and Gould, 2003; Kapur et al., 2012; Miller and Rockstroh, 2013). Genes involved in dopamine functioning may be directly involved in neuropsychiatric disorders (Gottesman and Gould, 2003; Insel, 2010; Miller and Rockstroh, 2013). In this case, a combined neurogenetic approach would be invaluable in the identification of endophenotypes—patterns of brain function that can be linked to a particular genotype (Gottesman and Gould, 2003; Insel, 2010; Miller and Rockstroh, 2013). The elucidation of genetic differences among patients may, for example, lead to improved understanding of diagnostic subtypes or creation of new subtypes (Charney et al., 2002).

An alternative, and perhaps more likely scenario is that the causative gene resides elsewhere but yet indirectly affect many related systems and circuits, including those mediated by dopaminergic genes (Insel et al., 2010; Papassotiropoulos and de Quervain, 2011). In this case, an understanding of the dopaminergic variation in genetically normal systems is no less valuable by facilitating understanding of therapeutic impacts (Charney et al., 2002). This is in particular if key defective genes identified prove to be difficult to target, in which case downstream genes or pathways affected by the illness that can be repaired constitutes a natural target of intervention (Wang et al., 2007; Chen et al., 2010; Yaspan and Veatch, 2011).

For some phenotypes of interest to social scientists, such as wealth or the aforementioned education attainment, the phenotype is sufficiently far removed from the underlying biology that little is gained by applying a pathway approach. In these cases, a purely exploratory GWAS approach may well be an appropriate choice. Even in these cases, however, exploratory versions of pathway analyses can be used. For example, “genome-wide pathway analysis” attempts to segment the genome in terms of biological processes and then attempts to find pathways differentially involved in a particular phenotype. This method has proved fruitful in identifying an association between IQ, a complex proxy-phenotype, and heterotrimeric G proteins that are central relay factors that may serve as “signaling bottleneck” for neuronal responses (Ruano et al., 2010). Another set of network-based methods uses graph theory methods to infer networks of genes that are involved in a phenotype, and are particularly useful for dealing with gene-gene interactions (Ramanan et al., 2012).

However, for a growing class of behavioral and clinical measures, the underlying biologically processes mapping sensory input to behavioral outcomes are increasingly mapped out at both neural and molecular levels. In these cases, pathways represent an important way of capturing our prior knowledge regarding biological processes mediating specific outcomes, and actionable therapeutic targets (Veenstra-VanderWeele and Anderson, 2000). Thus, if we think of a priori pathway selection as a “top-down” approach that generalizes the candidate gene approach, data-driven approaches can be thought of as a “bottom-up” approaches that generalizes the GWAS approach.

Overall, our approach explicitly acknowledges the inherent tension regarding our current state of knowledge (Robinson et al., 2008; Set et al., 2014). On the one hand, we now have an immense and growing base of knowledge regarding the biological basis of economic behavior, which can explain observation across multiple biological levels and, in some cases, across multiple species (Robinson et al., 2008; Connell and Hofmann, 2011). On the other hand, our knowledge is highly incomplete. For example, we still know little about the precise quantitative relationship between many of the allele variants in DA genes and gene expression levels, nor of their influence on neural circuits (Jia et al., 2011; Set et al., 2014). Finally, and perhaps most importantly, by centering the focus on biological processes as opposed to individual genes, a combined neurogenetic approach allows behavior across different studies to be related to a common set of mathematical principles, thereby moving beyond merely cataloging lists of genes and the myriad of associated behaviors.

### Conflict of interest statement

The authors declare that the research was conducted in the absence of any commercial or financial relationships that could be construed as a potential conflict of interest.
